# Residential Meditation Retreats: A Promise of Sustainable Well-Being?

**DOI:** 10.7759/cureus.73326

**Published:** 2024-11-09

**Authors:** Selvaraj Giridharan

**Affiliations:** 1 Oncology, Tawam Hospital, Al Ain, ARE

**Keywords:** inflammation, meditation, meditation retreats, mindfulness, qol, retreats, well-being, wellness and resilience

## Abstract

Meditation retreats are structured programs that immerse participants in focused meditation, mindfulness, and self-reflection over extended periods. Unlike conventional vacations, which prioritize relaxation, meditation retreats combine intensive practice with intentional rest, cultivating emotional regulation, mindfulness, and personal growth. These retreats have grown in popularity for their ability to support mental, emotional, and physical well-being. Research shows that retreats can reduce stress, anxiety, and depression while enhancing emotional resilience. Physical benefits, including reduced inflammatory markers and improved metabolic health, further contribute to long-term well-being. Positioned within the expanding wellness tourism market, meditation retreats offer sustainable benefits that surpass those of traditional leisure activities, marking them as a promising tool in preventive healthcare. In this editorial, we examine the evidence supporting these benefits and discuss challenges, such as varied formats, limited follow-up, and accessibility issues, which limit broader application. Further research is essential to standardise protocols, evaluate cost-effectiveness, and expand access, underscoring the potential of meditation retreats as a sustainable well-being intervention in today’s demanding world.

## Editorial

Introduction

Meditation has long been recognised for its capacity to promote mental, emotional, and physical well-being [1]. Rooted in ancient contemplative traditions and increasingly incorporated into modern wellness paradigms, meditation provides an effective method for stress reduction, emotional regulation, and personal development. In recent years, residential meditation retreats have emerged as specialised interventions that offer participants immersive environments to engage in sustained practice over extended periods of time.

These retreats deliver sustainable benefits by incorporating intensive, structured activities such as meditation, yoga, and silence. Participants reside in serene and distraction-free environments that facilitate introspection and cultivate emotional regulation. As part of the expanding wellness tourism market, valued at US$ 814.6 billion in 2022 and projected to grow at an annual rate of 12.42% by 2030, meditation retreats integrate relaxation with therapeutic practices [2]. This synthesis of mindfulness and self-care provides benefits beyond those of typical vacations, rendering retreats particularly valuable for individuals managing chronic conditions and aligning them with preventive healthcare initiatives [3].

Benefits of meditation retreats

Psychological Benefits

Several systematic reviews have elucidated the psychological benefits of meditation retreats. Naidoo et al. and Khoury et al. reported significant reductions in anxiety, depression, and stress, with novice meditators exhibiting the most substantial improvements in emotional well-being [4,5]. These findings suggest that the intensive nature of retreats facilitates emotional regulation, even in individuals with limited prior mindfulness experiences. Central to these psychological improvements is the cultivation of mindfulness, which enables participants to regulate their emotions and effectively navigate challenges.

Studies have further demonstrated that mindfulness gains account for up to 50% of the psychological improvements achieved through retreats, underscoring the significance of immersive practices in developing resilience and emotional stability. McClintock et al. corroborated these findings, with reductions in anxiety and depression persisting several weeks post retreat, illustrating the enduring psychological benefits of intensive meditation [6].

Physical Health Benefits

Meditation retreats have demonstrated efficacy in improving physical health and metabolic function. A study from an advanced meditation program reported reductions in C-reactive protein (CRP) levels, improved lipid profiles, and a 3% reduction in body weight over the course of the retreat [7]. These outcomes elucidate the capacity of retreats to support the control of systemic inflammation and promote healthy weight management through mindfulness and physical practice.

Similarly, a three-day mindfulness retreat study conducted by Gardi et al. demonstrated reductions in the pro-inflammatory cytokines IL-6 and IL-8, along with an increase in the anti-inflammatory cytokine IL-10 [8]. These physiological changes suggest that meditation positively influences immune regulation, and potentially mitigates the effects of chronic stress and inflammation. Additionally, participants exhibited a reduction in cortisol levels, which correlated with decreases in anxiety and perceived stress, underscoring the role of meditation in fostering physiological resilience.

Long-Term Well-Being and Cognitive Benefits

In contrast to vacations, whose positive effects often diminish within a few weeks, the benefits of meditation retreats appear to be more enduring. Blasche et al. found that participants experienced sustained improvements in emotional regulation and fatigue reduction for up to 10 weeks post retreat [9]. In addition to emotional well-being, meditation retreats enhanced cognitive function. King et al. identified improvements in sustained attention and emotional regulation, along with increased telomere length, a marker associated with healthy ageing [10]. These findings emphasise the potential of meditation to foster long-term cognitive and physiological well-being, thus reinforcing the broader health benefits of immersive retreat experiences.

The reviewed studies consistently demonstrated that meditation retreats confer substantial and enduring benefits that exceed those of vacations or outpatient mindfulness programs [9]. These retreats provide psychological relief by significantly reducing stress, anxiety, and depression while fostering emotional regulation and resilience. The participants developed mindfulness skills that enhanced their capacity to manage emotional challenges, contributing to sustained improvements in well-being [10].

In addition to mental health benefits, retreats have shown significant physical health improvements, including the reduction of inflammatory markers such as CRP, IL-6, and IL-8, as well as enhancements in metabolic health, specifically improvements in lipid profiles, blood glucose regulation, and body weight reduction [7,8]. These physiological changes reflect the potential of mediation to regulate immune function and mitigate the effects of chronic stress. These findings also suggest that retreats can play a pivotal role in preventive healthcare, promoting long-term health, and reducing the risk of chronic diseases.

Meditation retreats provide a structured environment that is conducive to personal growth, mindfulness, and self-care. This intentional immersion allows participants to cultivate practices that extend beyond the retreat, thereby promoting habits that contribute to sustainable well-being [6]. The skills acquired during these experiences, such as mindfulness, emotional regulation, and healthy lifestyle practices, continue to benefit participants long after the retreat concludes, rendering these programs as valuable tools for long-term well-being [11].

Challenges and future directions

Despite compelling evidence, several challenges impede the broader application and acceptance of meditation retreats. A primary issue is the heterogeneity in retreat formats, ranging from silent meditation programs such as Vipassana to more integrative wellness retreats that incorporate yoga and dietary interventions. This variability complicates comparisons across studies and limits the development of standardised protocols for assessing outcomes [5].

Another limitation is the short follow-up periods prevalent in retreat studies, making it difficult to evaluate the long-term sustainability of psychological and physical benefits [4]. Further longitudinal research is necessary to determine whether improvements in emotional regulation, mindfulness, and health markers persist for several weeks or months. The limited generalisability of our findings presents a challenge. Retreat participants tended to self-select, often entering programmes with higher levels of motivation or baseline mindfulness. This introduces bias and reduces the applicability of the results to broader populations, including individuals with limited prior meditation experience or severe mental health challenges.

Moreover, costs and accessibility are significant barriers. Many retreats are time-intensive and expensive, rendering them accessible primarily to privileged individuals. Without addressing these financial and logistical challenges, it will be difficult for meditation retreats to become viable and inclusive tools in preventive healthcare. Additional research on the economic impact and cost-effectiveness of these programs is required to position them as accessible non-pharmacological interventions within healthcare frameworks.

Conclusion

Meditation retreats provide an effective intervention for addressing the stress and distractions of modern life. By combining intensive practice with intentional rest, they offer substantial psychological and physical health benefits. Unlike conventional vacations, which provide only temporary relief, retreats cultivate lasting mindfulness, emotional regulation, and personal growth. Evidence suggests that these programs promote sustainable well-being by embedding self-care practices that benefit participants long after the retreat ends. As wellness tourism grows, meditation retreats have the potential to become an essential component of preventive healthcare, offering accessible, non-pharmacological strategies for enhancing mental and physical health.

## References

[1] Schlechta Portella CF, Ghelman R, Abdala V, Schveitzer MC, Afonso RF (2021). Meditation: evidence map of systematic reviews. Front Public Health.

[2] Summit GW (2024). Global Wellness Summit. 2020 global wellness trends. https://www.globalwellnesssummit.com/2020-global-wellness-trends/.

[3] Norman A, Pokorny JJ (2017). Meditation retreats: spiritual tourism well-being interventions. Tour Manag Perspect.

[4] Naidoo D, Schembri A, Cohen M (2018). The health impact of residential retreats: a systematic review. BMC Complement Altern Med.

[5] Khoury B, Knäuper B, Schlosser M, Carrière K, Chiesa A (2017). Effectiveness of traditional meditation retreats: a systematic review and meta-analysis. J Psychosom Res.

[6] McClintock AS, Rodriguez MA, Zerubavel N (2019). The effects of mindfulness retreats on the psychological health of non-clinical adults: a meta-analysis. Mindfulness.

[7] Sadhasivam S, Alankar S, Maturi R (2021). Isha yoga practices and participation in Samyama program are associated with reduced HbA1c and systemic inflammation, improved lipid profile, and short-term and sustained improvement in mental health: a prospective observational study of meditators. Front Psychol.

[8] Gardi C, Fazia T, Stringa B, Giommi F (2022). A short mindfulness retreat can improve biological markers of stress and inflammation. Psychoneuroendocrinology.

[9] Blasche G, deBloom J, Chang A, Pichlhoefer O (2021). Is a meditation retreat the better vacation? Effect of retreats and vacations on fatigue, emotional well-being, and acting with awareness. PLoS One.

[10] King BG, Conklin QA, Zanesco AP, Saron CD (2019). Residential meditation retreats: their role in contemplative practice and significance for psychological research. Curr Opin Psychol.

[11] Cohen JN, Jensen D, Stange JP, Neuburger M, Heimberg RG (2017). The immediate and long-term effects of an intensive meditation retreat. Mindfulness.

